# Development of new measurement system of thoracic excursion with biofeedback: reliability and validity

**DOI:** 10.1186/1743-0003-10-45

**Published:** 2013-05-20

**Authors:** Yukiko Nishigaki, Hiroko Mizuguchi, Eriko Takeda, Tomokazu Koike, Takeshi Ando, Kazuya Kawamura, Takuro Shimbo, Hidetoshi Ishikawa, Masashi Fujimoto, Ikuko Saotome, Reiko Odo, Kazuko Omoda, Shohei Yamashita, Tomoko Yamada, Toshihito Omi, Yuya Matsushita, Manami Takeda, Sawako Sekiguchi, Saki Tanaka, Masakatsu Fujie, Haruhi Inokuchi, Junko Fujitani

**Affiliations:** 1 Department of Rehabilitation, National Center for Global Health and Medicine, 1-21-1 Toyama, Shinjuku-ku, Tokyo 162-8655, Japan; 2 Graduated School of Medicine, Osaka University, 1-7 Yamadaoka, Suita, Osaka 565-0781, Japan; 3 Faculty of Science and Engineering, Waseda University, 3-4-1 Ohkubo, Shinjyuku-ku, Tokyo 169-8555, Japan; 4 Department of Clinical Research and Informatics, National Center for Global Health and Medicine, 1-21-1 Toyama, Shinjuku-ku, Tokyo 162-8655, Japan; 5 Department of Rehabilitation Medicine, Graduate School of Medicine, The University of Tokyo, 7-3-1 Hongo, Bunkyo-ku, Tokyo 113-8655, Japan

**Keywords:** Respiratory rehabilitation, Chest expansion score, Biofeedback, Reliability

## Abstract

**Background:**

Respiratory rehabilitation reduces breathlessness from patient with respiratory dysfunction. Chest expansion score, which represents the circumference magnitude of the thoracic cage, is used for a target when treating patients with respiratory disease. However, it is often difficult for patients to understand the changes in the respiratory status and be motivated for therapy continuously. We developed a new measurement system with biofeedback named BREATH which shows chest expansion scores in real time. The purpose of this study was to determine the reliability and validity of the novel system in advance of clinical application.

**Methods:**

Three evaluators measured chest expansion in 33 healthy individuals using tape measure, which is used for the measurement traditionally, and BREATH. The wire for BREATH system was threaded over the thoracic continuously and the data was recorded automatically; whereas the tape was winded and measured each maximal expiration and inspiration timing by evaluator. All participants were performed both measurement simultaneously for three times during deep breath. In this study, we studied chest expansion score without using biofeedback data of BREATH to check the validity of the result. To confirm intra- and inter-evaluator reliability, we computed intra-class correlations (ICCs). We used Pearson’s correlation coefficient to evaluate the validity of measurement result by BREATH with reference to the tape measure results.

**Results:**

The average (standard deviation) chest expansion scores for all, men and women by the tape measure were 5.53 (1.88), 6.40 (1.69) and 5.22 (1.39) cm, respectively, and those by BREATH were 3.89 (2.04), 4.36 (1.83) and 2.89 (1.66) cm, respectively. ICC within and among the three evaluators for BREATH and the tape measure were 0.90-0.94 and 0.85-0.94 and 0.85 and 0.82, respectively. The correlation coefficient between the two methods was 0.76-0.87.

**Conclusion:**

The novel measurement system, BREATH, has high intra- and inter-evaluator reliabilities and validity; therefore it can lead us more effective respiratory exercise. Using its biofeedback data, this system may help patients with respiratory disease to do exercises more efficiently and clinicians to assess the respiratory exercise more accurately.

## Background

Respiratory rehabilitation reduces breathlessness from patients with respiratory dysfunction; i.e. chronic obstructive pulmonary disease (COPD) and postoperative respiratory dysfunction. In COPD patients, flat diaphragm and overexpanded lungs reduce the expansion efficiency of lower chest. Breathing muscle stretching exercise improves chest expansion ability and pulmonary function [1].

We measure chest expansion to assess the effect of treatment for patient with respiratory disease. There are some devices to measure chest expansion; i.e. spirometry or respiratory inductive plethysmograph, which can measure both chest and abdominal expansion [2]. In Japanese guidelines for pulmonary rehabilitation, the chest expansion scores, representing the thoracic cage movement while breathing, are described as a standardized evaluation for the exercise of respiratory ailments [3]. The score represents the circumference magnitude of the thoracic cage from maximum inhalation to maximum exhalation, which is measured by tape traditionally [3-5]. It is known that the chest expansion score of patient with COPD at the level of 10^th^ rib height significantly improved after chest mobilization [6].

Biofeedback is known to be useful for re-education of the dysfunctional muscles. Past study shows that it is effective to perform respiratory rehabilitation with biofeedback to strengthen the muscles which control breathing [7-9]. Other studies suggest that respiratory rehabilitation with biofeedback helped ventilator weaning for patients with various disease [10-13].

Tape measurement of the chest expansion is generally performed during exercise in research or clinic, but it shows only temporary numerical value, which is difficult for subject or patient to interpret. Recently, new techniques for measuring the motion of the thoracic cage with biofeedback data have been developed [14,15]; however, it is often difficult for subjects or patients to understand changes in the respiratory status and be motivated for therapy continuously by the data. Thus, to show clear-cut biofeedback data will become feasible for them if the differences in thoracic expansion is measured and visualized in real time with uncomplicated technique.

We developed a new measurement system with biofeedback, which displays chest expansion scores simply in real time [16]. The purpose of the present study was to determine the reliability and validity of the novel system in advance of clinical application.

## Methods

### Chest expansion measurement device (BREATH)

**Figure 1:**
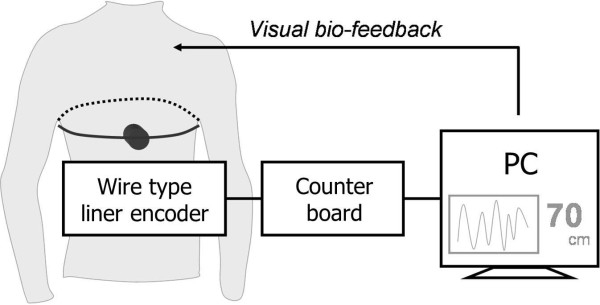
**Schema of our novel system and a measurement scenario.** To measure the magnitude of chest circumference, we use a wire-type linear encoder to wrap around the thoracic cage. The wire changes its length automatically to fit with the thoracic cage. The encoder detects the displacement length of wire over time, and the counter board transverses wire length to numerical data. The data is sent to a connected personal computer.

**Figure 2:**
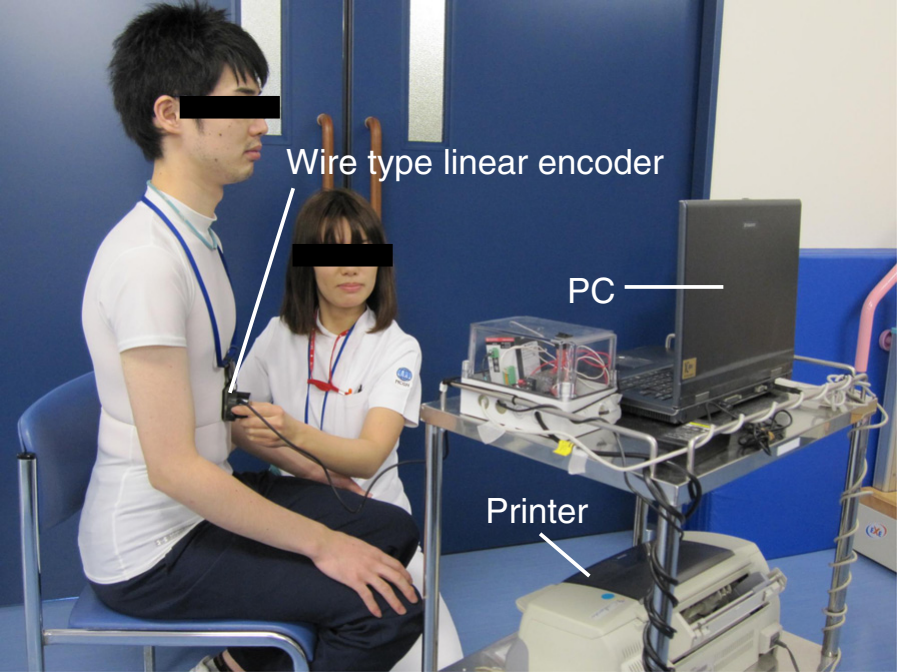
**Scenary of measurement.** Participant was asked to wear T-shirt and sit comfortably. The wire was threaded over the thoracic continuously, whereas the tape was winded and measured each maximal expiration and inspiration timing. We placed the wire and the tape over the 10^th^ rib edge to the sternum and wrapped them around the trunk horizontally.

**Figure 3:**
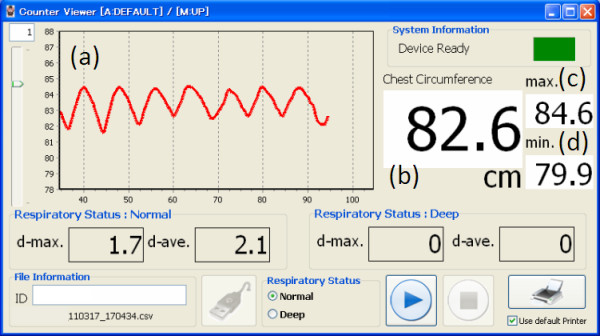
**Display of the PC.** The personal computer monitor displays (**a**) trend graphs of chest expansion score over about 10 past breaths, and the circumference (**b**) in real time (cm), (**c**) at the maximal (cm), and (**d**) at the minimal (cm).

Prior to the research, we had developed a novel system to measure the thoracic circumference, named BREATH [17]. Figure 1 shows a schema of our novel system and a measurement scenario. To measure the magnitude of chest circumference, we use a wire-type linear encoder to wrap around the thoracic cage (Figure 2). The wire changes its length automatically to fit with the thoracic cage. The encoder detects the displacement length of wire over time, and the counter board transverses wire length to numerical data. The data is sent to a connected personal computer (PC), whose monitor displays chest circumferences and a trend of chest expansion scores over about 10 past breaths, which is the expanded length from minimum circumference (Figure 3).

### Ethical considerations

The Institutional Review Board of our center had approved the study protocol. Each participant gave written informed consent prior to the study.

### Subjects

Healthy individuals from 20 to 60 years old with no history of lung or locomotor diseases were recruited for the study.

### Measurement procedure

We measured the thoracic circumference by tape and BREATH. Participant was asked to wear a T-shirt and to seat comfortably. We chose tape measure as a comparison because it has been widely used in a clinical situation and can conduct simultaneously with BREARH measurement to check the validity. The wire was threaded over the thoracic continuously, whereas the tape was winded and measured each maximal expiration and inspiration timing. We placed the wire and the tape over the 10^th^ rib edge to the sternum and wrapped them around the trunk horizontally. The height was chosen in regards of the past study result which showed the measurement result from the height had higher clinical value [6].

We randomized the order of two measurement methods; 16 participants underwent BREATH first, and the other 17 did the tape measure first. Before starting the measurement, all participants were instructed to “breathe as deeply as possible” and practiced to breath for several times. To check the validity of result from BREATH, they were blinded to the results of the test. The interval between each evaluation was at least 5 minutes to minimize participant’s fatigue among the study.

Three evaluators repeated measurements three times using both tape measurement and BREATH to all participants. Evaluator A had 6 years of experience as a physical therapist, and evaluators B and C had 2 and 10 years of experience, as occupational therapists, respectively. Each evaluator calculated and recorded chest expansion scores by tape measure data and by BREATH data. The evaluators were blinded to one another’s results.

### Statistical analysis

We used two-sample t test to compare variables of men with those of women. To confirm the intra-evaluator reliability of the measurement result by each evaluator using tape measure and BREATH, the intra-class correlation coefficient (ICC) was computed. Similarly, the inter-evaluator reliability among the measurement results by all three evaluators was evaluated using ICC, computed from the average data of each evaluator. ICCs of >0.9, 0.8-0.9, 0.7-0.8, 0.6-0.7 and <0.6 were considered excellent, good, acceptable, marginal and unreliable, respectively [18].

The validity of measurement result by BREATH was evaluated using Pearson’s correlation coefficient with reference to the tape measure results. To visualize the validity, we also constructed a Bland-Altman plot in which the y axis showed the difference between both measurements and the x axis showed the average of both measurements [19]. For all test, a p value < 0.05 was considered significant. All data were analyzed using IBM SPSS Statistics ver.19.

## Results

Thirty-three healthy participants (13 men and 20 women, age 29.2 years old, body height 166.6 cm, body weight 59.6 kg, body mass index (BMI) 21.4 kg/m^2^ in average) enrolled this study (Table 1). Body height, body weight, and BMI were significantly higher for men than for women.

**Table 1:** Characteristics of the participants

	**All (n = 33)**	**Men (n = 13)**	**Women (n = 20)**	p value*
Age (y)	29.2 ± 7.7	28.1 ± 8.1	29.9 ± 7.6	0.52
Height (cm)	166.6 ± 8.2	173.3 ± 6.4	162.2 ± 6.0	<0.0001
Weight (kg)	59.6 ± 11.5	68.0 ± 10.7	54.2 ± 8.3	0.0002
BMI (kg/m^2^)	21.4 ± 2.94	22.6 ± 3.30	20.5 ± 2.42	0.042

Average ± standard deviation. *BMI*, body mass index. *, Test of significance between men and women: two sample t-test.

### Chest expansion scores

All participant completed trials and all evaluator finished measurement without any deficit. The average (standard deviation: SD) chest expansion scores for all, men and women by the tape measure were 5.53 (1.88), 6.40 (1.69) and 5.22 (1.39) cm, respectively, and those by BREATH were 3.89 (2.04), 4.36 (1.83) and 2.89 (1.66) cm, respectively (Table 2). Chest expansion score was significantly higher for men than for women in both measurements.

**Table 2:** Average chest expansion scores from three evaluators

	**All (n = 99)**	**Men (n = 39)**	**Women (n = 60)**	p value*
Tape measure (cm)	5.53 ± 1.88	6.56 ± 1.80	4.86 ± 1.61	<0.0001
BREATH (cm)	3.89 ± 2.04	5.00 ± 1.87	3.16 ± 1.82	<0.0001

Average ± standard deviation. *, Test of significance between men and women: two sample t-test.

### Intra- evaluator reliability

All evaluators showed similar results for either method (Table 3). The ICCs for the three evaluators ranged from 0.90 to 0.94 for BREATH and from 0.85 to 0.94 for the tape measure (Table 4). There were no apparent correlations between years of experience and the reliabilities for either method.

**Table 3:** Average chest expansion scores by each evaluator

	**Evaluator**		
	**A**	**B**	**C**
BREATH (cm)	3.46 ± 0.51	4.05 ± 0.44	4.14 ± 0.57
Tape measure (cm)	5.68 ± 0.51	5.44 ± 0.49	5.47 ± 0.62

Average ± standard deviation.

**Table 4:** Intra-evaluator reliability of BREATH and tape measure

	**Evaluator**		
	**A**	**B**	**C**
BREATH	0.90 (0.82-0.94)	0.94 (0.90-0.97)	0.90 (0.83-0.95)
Tape measure	0.87 (0.78-0.93)	0.94 (0.90-0.97)	0.85 (0.75-0.92)

Inter-class correlation (95% confidence interval).

### Inter- evaluator reliability

The result from evaluator B, whose clinical experience was shorter than other two, did not differ from results from others. The ICCs among the three evaluators were 0.85 (95% confidence interval: 0.70-0.90) for BREATH and 0.82 (95% confidence interval: 0.73-0.92) for the tape measure, indicating that both measurement techniques were reliable.

### Validity of BREATH compared with the tape measure

**Figure 4:**
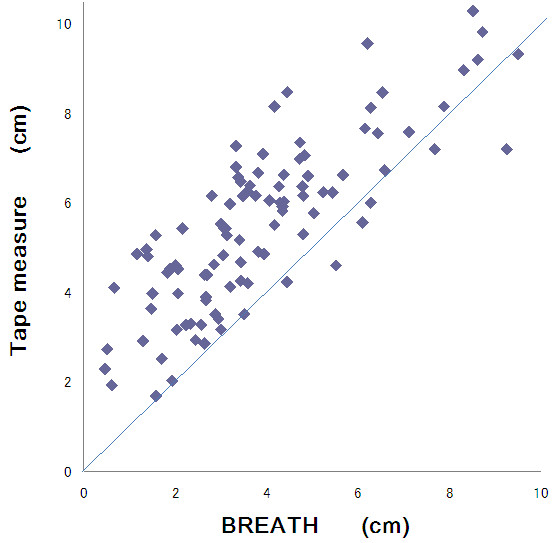
**Scatterplots of chest expansion scores by BREATH and tape measure.** Each dot shows data of each participant by each evaluator. Pearson’s correlation coefficients for the measurement methods were 0.76-0.87 for the three evaluators, which confirmed high validity of BREATH compared with the tape measure (p < 0.001).

**Figure 5:**
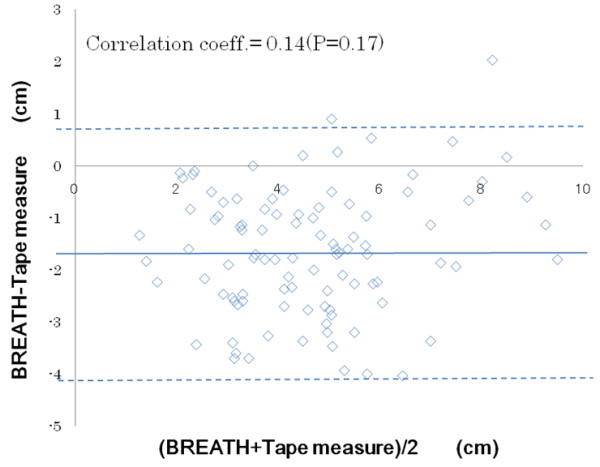
**Bland-altman plot for rape measurement and BREATH.** Each dot shows data of each participant by each evaluator. The total the average (SD) of differences between both measurement was -1.65 (1.21) cm. The averages and the difference of the chest expansion scores were not significant (correlation coefficient 0.139, p = 0.169). There was no bias to make gradient.

Pearson’s correlation coefficients for the measurement methods were 0.76-0.87 for the three evaluators, which confirmed high validity of BREATH compared with the rape measure (p < 0.001, Figure 4). Figure 5 shows that the average (SD) difference between both measurement was -1.65 (1.21) cm and that the difference of the chest expansion scores was not significant (correlation coefficient 0.139, p = 0.169). There was no bias to make gradient in the plot.

## Discussion

We developed the novel BREATH system that displays chest expansion scores in real time [17], and, thus, renders feasible training by visual biofeedback. Moreover, tasks such as calibration, measurement start and stop, and printing results can be easily performed with only three mouse clicks, which allow us to use it in a clinical setting. We got high reproducibility and reliability of BREATH and high validity compared with the conventional tape measure method. Though, there are some other devices to measure respiratory function [2], they have no visible biofeedback information. With its high validity as a measurement system and with its biofeedback data, BREATH would also help to increase efficacy of respiratory rehabilitation.

Intra- evaluator reliability for BREATH and tape measure was high, with ICC values of ≥0.8. The reproducibility and reliability of automated measurements taken by BREATH were higher than those obtained manually by the tape measure. We presume the high reliability of BREATH is due to the location invariance of the wire compared to the tape. As the tape was removed between each measurement, the position might be changed; whereas wire for BREATH was remained during all measurement. Therefore, we believe that BREARH can measure chest expansion score accurately regardless of the evaluator’s work experience.

We suggest that the chest circumference measured by BREATH describes more precise data than tape measurement. Although the values obtained by BREATH and the tape measure closely correlated, those by BREATH were lower with an average of -1.65 cm. We hypothesize the wire used in BREATH system fits more tightly to participant’s body than the tape used in the conventional method; therefore, it served smaller numerical values. The difference should be taken into account when we compare the results from both methods.

We think we should use chest expansion score from men and women data separately. In both measurements, men showed higher score than women. We presume that men have lager rib cage and larger magnitude than women.

We confirmed that our measurement system was feasible and, thus, chest expansion training would be more effective by using the system with biofeedback. We need further study using biofeedback system because we did not give biofeedback data for the participants in this study. The present results suggest a promising future for chest measurement system with biofeedback in respiratory rehabilitation. It would be important to compare BREATH and other measurement for respiratory function. Other limitation of the present study is that we included only healthy adults as participants and excluded elderly persons and patients with thoracic cage abnormalities. We should confirm the accuracy of measurement for such individuals before we use BREATH for routine clinical applications.

## Conclusion

The novel biofeedback technology, BREATH, is reliable and valid and can lead us more effective respiratory exercise. This system may help patients with respiratory disease to do exercises more efficiently and clinicians to assess the respiratory exercise more accurately.

## Competing interests

All authors declare that they have no competing interests.

## Authors’ contributions

YN participated in the design of the study, recruited the participants, managed acquisition of data, and drafted the manuscript. HM, ET and TK assisted in the design of the study, recruited the participants, managed acquisition of data. TA, KK and MF participated in the development of device. TS participated in data analysis and drafted the manuscript. HI, MF, IS, RO, KO, SY, TO, YM, MK, SK, ST and MF participated in managed acquisition of data. JF and HI participated in the design of the study, and drafted the manuscript. All authors read and approved the final manuscript.
